# Prognostic significance of inflammatory and nutritional indicators for treatment outcomes in untreated tuberculosis patients with hypertension

**DOI:** 10.3389/fnut.2026.1801389

**Published:** 2026-04-22

**Authors:** Sifang Xiao, Anhua Cao, Yiping Leng

**Affiliations:** 1 Department of Laboratory, The Affiliated Changsha Central Hospital, Hengyang Medical School, University of South China, Changsha, Hunan, China; 2 Central Laboratory, Affiliated Haikou Hospital of Xiangya Medical College, Central South University, Haikou, China; 3 Department of Cardiology, The Affiliated Changsha Central Hospital, Hengyang Medical School, University of South China, Changsha, Hunan, China; 4 Changsha Tuberculosis Research Institute, The Affiliated Changsha Central Hospital, Hengyang Medical School, University of South China, Changsha, Hunan, China; 5 Central Laboratory, The Affiliated Changsha Central Hospital, Hengyang Medical School, University of South China, Changsha, Hunan, China

**Keywords:** hypertension, inflammation, nutrition, pulmonary tuberculosis, treatment outcomes

## Abstract

**Objective:**

Inflammation and malnutrition play pivotal roles in disease progression. This study evaluated the associations between inflammation/nutrition-based composite indices and treatment outcomes in hospitalized adults with previously untreated pulmonary tuberculosis (PTB) complicated by hypertension.

**Methods:**

We included hospitalized adults with untreated PTB and concomitant hypertension admitted to Changsha Central Hospital (from January 2019 to December 2021). Inflammation/nutrition-based composite indices including Prognostic Nutritional Index (PNI), Hemoglobin-Albumin-Lymphocyte-Platelet (HALP) score, Monocyte-to-Albumin Ratio (MAR), Neutrophil-to-Albumin Ratio (NAR), and Red Cell Distribution Width-to-Albumin Ratio (RAR) were calculated. Associations between these indices and PTB treatment outcomes were evaluated using multivariable logistic regression, restricted cubic splines, and subgroup analysis.

**Results:**

Among 1,012 hospitalized patients with untreated PTB complicated by hypertension, 166 individuals (16.40%) experienced unfavorable treatment outcomes. After multivariate adjustment, logistic regression revealed that RAR (aOR 0.75, 95% CI 0.64–0.89, *p* = 0.001) and NAR (aOR 0.40, 95% CI 0.19–0.84, *p* = 0.015) were inversely associated with favorable outcomes, while PNI was positively associated (aOR 1.03, 95% CI 1.01–1.06, *p* = 0.011). Restricted cubic splines revealed a negative linear association for NAR (*p* = 0.030) and RAR (*p* = 0.013), and a positive linear association for PNI (*p* = 0.002). Machine learning algorithms including GLM, RF, and SVM ranked the relative contributions of inflammation/nutrition-based indices and their components to treatment outcomes, with RAR identified as the top-contributing indicators.

**Conclusion:**

Routine laboratory-derived composite indices, particularly RAR and PNI, were associated with treatment outcomes in previously untreated PTB patients with hypertension, underscoring the importance of baseline nutritional and inflammatory status in the management of this patient population.

## Introduction

Tuberculosis (TB) is an infectious disease caused by *Mycobacterium tuberculosis* and remains one of the major global public health threats (1, 2). According to the World Health Organization (WHO) Global Tuberculosis Report 2024, there were approximately 10.8 million new TB cases worldwide in 2023 and about 1.25 million TB-related deaths (including HIV-related deaths) (3), ranking TB among the leading single-disease causes of mortality (4). Despite the United Nations Sustainable Development Goals’ commitment to ending the TB epidemic by 2030, diagnostic delays, poor treatment adherence, and the transmission of drug-resistant strains persist (5, 6), underscoring the urgent need to strengthen interventions to reduce transmission and improve outcomes (7).

Hypertension, as a common chronic non-communicable disease, has a relatively high comorbidity rate among TB patients (8). Existing studies indicate that hypertension can influence the pathological course of TB by inducing systemic inflammation and thereby increase all-cause mortality and infection-related mortality in TB patients (9, 10). Moreover, malnutrition and inflammatory states are key factors affecting TB treatment outcomes (11, 12). We investigated whether these inflammation- and nutrition-based indicators are associated with treatment outcomes among hospitalized patients with tuberculosis and hypertension, providing clinical recommendations for future efforts to control tuberculosis mortality and achieve the WHO “End TB” strategy.

Numerous studies have focused on the comorbidity of TB and hypertension. For example, a cohort study found that patients with hypertension with TB had a significantly higher all-cause mortality risk within nine months after treatment (9); another study reported an approximate 64% increase in mortality risk over a two-year follow-up period in such patients (10). The use of calcium channel blockers may improve survival in patients with hypertension and TB (9), yet the systemic impact of nutritional-inflammatory status on this population has not been comprehensively evaluated. Nutritional-inflammatory indices such as the Prognostic Nutritional Index (PNI) have been demonstrated to be prognostic in cancer and cardiovascular diseases and have shown value in predicting mortality risk among TB patients (13). However, other emerging indices, including the Hemoglobin-Albumin-Lymphocyte-Platelet (HALP), Monocyte-Albumin Ratio (MAR), Neutrophil-Albumin Ratio (NAR), and Red Cell Distribution Width-Albumin Ratio (RAR), have limited application in the TB field. Although the interactions between hypertension and TB have been partially elucidated, there remains a lack of comprehensive multi-indicator studies focused on TB patients with hypertension, particularly regarding the associations of these nutritional-inflammatory indices with treatment outcomes.

This study aims to explore the associations between nutritional-inflammatory indices, including HALP, MAR, NAR, RAR, and PNI, and treatment outcomes in patients with newly diagnosed tuberculosis coexisting with hypertension. By integrating clinical data with multi-index analyses, the study seeks to provide evidence for early identification of high-risk patients and the optimization of individualized treatment strategies, thereby filling the gap in nutritional-inflammatory assessment within the comanagement of tuberculosis and hypertension. The findings may offer a theoretical basis for targeted interventions (such as nutritional support or anti-inflammatory therapy) to improve prognosis in this population.

## Materials and methods

### Study population

**Figure 1:**
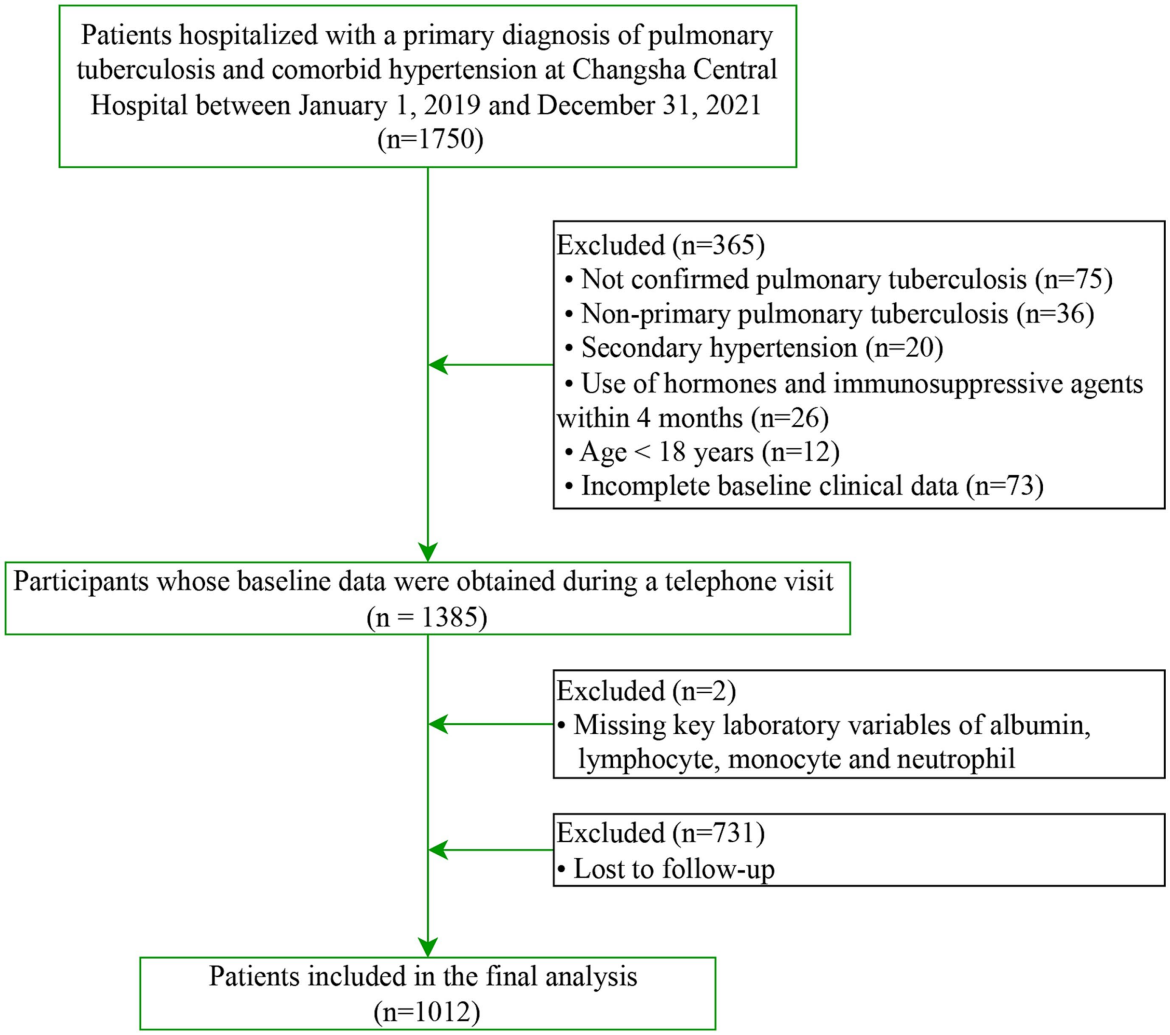
The flowchart of this study.

This study represents a secondary analysis of a previously established retrospective cohort comprising adult patients with initially treated pulmonary tuberculosis (PTB) complicated by hypertension, who were admitted to Changsha Central Hospital Affiliated with the University of South China between January 2019 and December 2021 (14). In accordance with national tuberculosis control guidelines in China (15), all patients received standardized care at designated institutions with corresponding medical insurance coverage. The inclusion criteria were as follows: (i) initially treated PTB, diagnosed according to the Chinese Diagnostic Criteria for Pulmonary Tuberculosis (WS 288—2017) based on a combination of clinical, imaging, and etiological evidence (smear, culture, or molecular tests). Following established classifications (14), this included patient who had (a) never received anti-tuberculosis therapy; (b) not completed a standard regimen, or (c) had irregular treatment for less than 1 month (operationally defined via medical records as physician-documented interrupted anti-tuberculosis therapy with a cumulative duration of less than 30 days); (ii) established an diagnosis of essential hypertension according to the 2018 Chinese Guidelines for the Management of Hypertension (16). Specifically, hypertension was defined as a clinic systolic blood pressure (SBP) ≥ 140 mmHg and/or diastolic blood pressure (DBP) ≥ 90 mmHg recorded on three different days in the absence of antihypertensive medications, or the current use of antihypertensive therapy for a documented history of hypertension even if the presenting blood pressure was < 140/90 mmHg; (iii) age ≥ 18 years; (iv) non-pregnant or non-lactating status; (v) Individuals without conditions that compromise immune function, such as HIV infection, active malignancy, hematologic disorders, systemic lupus erythematosus, rheumatoid arthritis, or a history of organ transplantation; (vi) no use of glucocorticoids or immunosuppressive agents in the preceding 4 months; and (vii) availability of complete baseline clinical and laboratory data. The process of participant selection is illustrated in Figure 1.

The original study was conducted in accordance with the Declaration of Helsinki. As a retrospective cohort study utilizing anonymized data from the hospital’s electronic medical record system, it was granted exemption from informed consent. Ethical approval was obtained from the Medical Ethics Sub-committee of Changsha Central Hospital (Ethics Approval Number: 2023-049).

### Assessment of inflammation and nutritional indicators

Routine laboratory tests included serum albumin (ALB) levels along with hematological parameters such as hemoglobin (HGB), platelet count, red blood cell distribution width (RDW), and absolute counts for neutrophils, lymphocytes, and monocytes. Testing methods and reference ranges are provided in Supplementary Table 1. Using the aforementioned hematological and biochemical parameters related to inflammation and nutrition, we calculated several composite indices that integrated both nutritional status and inflammatory response. The derived inflammation-nutrition markers comprised the NAR, MAR, RAR, prognostic nutritional index (PNI), and HALP score (17–21). Each of these composite indices was computed through specific combinations of the individual parameters described above, with detailed formulas presented in Supplementary Table 2.

### Assessment of outcomes

Tuberculosis treatment outcomes were categorized as either favorable or unfavorable (22). Outcome assessment was conducted 12 months after the last patient was discharged, through electronic medical record review and telephone follow-up. Favorable outcomes were defined as achieving either clinical cure or bacteriological cure, as specified in the national TB treatment guidelines (15). Unfavorable outcomes included any of the following: treatment failure, disease recurrence, development of drug resistance, or death (whether TB-related or non-TB related) (22). Treatment failure was defined according to the Guidelines for Primary Diagnosis and Treatment of Pulmonary Tuberculosis (23) as persistent sputum smear positivity in smear-positive patients at month 6 or at treatment completion. Recurrence was defined as the reappearance of active tuberculosis after completion of therapy and achievement of cure or treatment completion during the follow-up period (24, 25). Drug resistance was determined by microbiological drug-susceptibility testing (DST) or molecular diagnostic methods as documented in the medical records, with drug-resistant tuberculosis defined as resistance to at least one first-line anti-tuberculosis drug confirmed by either phenotypic DST or molecular testing (26). Death was defined as all-cause mortality, including both tuberculosis-related and non-tuberculosis-related deaths occurring during anti-tuberculosis treatment or during the post-treatment follow-up period (14).

### Other covariates

In this study, the covariates were adjusted for a range of factors, including demographic characteristics, lifestyle-related factors, and clinical parameters. Demographic variables included age, sex, and education level (categorized as junior high school or below versus senior high school or above). Lifestyle factors encompassed smoking status and alcohol use. Concurrent medical conditions included diabetes mellitus (DM), coronary heart disease (CHD), chronic obstructive pulmonary disease (COPD), and chronic kidney disease (CKD), among other conditions. Additionally, baseline physiological measures included admission blood pressure and heart rate, hypertension duration, and duration of pulmonary tuberculosis. Sputum microbiology results (defined as positive when acid-fast bacilli smear or culture yielded positive findings), the presence of drug-resistant strains, and patterns of antihypertensive and lipid-modifying therapy were also recorded. Follow-up information encompassed the course of anti-tuberculosis therapy and patient adherence to prescribed regimens. Covariates with less than 10% missing data were handled using multiple imputation; those with more than 10% were excluded.

### Statistical analysis

Continuous variables were assessed for normality using histograms and the Kolmogorov–Smirnov test. Normally distributed variables are presented as mean ± standard deviation (SD), and non-normally distributed variables are presented as median (interquartile range, IQR). Categorical variables are expressed as counts and proportions. Between-group differences were compared using the independent samples t-test for normally distributed continuous variables, the Mann–Whitney U test for non-normally distributed continuous variables, and the Pearson chi-squared test or Fisher’s exact test for categorical variables as appropriate.

Multivariable logistic regression was utilized to explore links between nutrition- and inflammation-related markers and outcomes of TB therapy. Three models were built progressively: a crude model without adjustment; Model 1 controlling for age and gender; and Model 2 extending adjustments to include education, smoking, alcohol intake, previous stroke, CHD, CKD, COPD, hyperlipidemia, and diabetes. The selection of covariates relied on clinical experience, availability, and prior literature (27, 28). Model goodness of fit was assessed using the Akaike Information Criterion (AIC) and the Hosmer-Lemeshow test. To further examine whether these associations followed a dose–response pattern, potential non-linear associations of the indicators (HALP, MAR, NAR, RAR, and PNI) with TB therapy results in untreated tuberculosis cases accompanied by hypertension were investigated via restricted cubic spline (RCS) regression employing three knots located at the 10th, 50th, and 90th percentiles based on the Bayesian Information Criterion (BIC).

To assess the robustness of the association, we performed subgroup analysis. We examined the relationships between RAR, HALP, NAR, MAR, and PNI and treatment outcomes according to age (< 60 years vs. ≥ 60 years), gender (male vs. female), smoking (no vs. yes), drinking (no vs. yes), diabetes (no vs. yes), CKD (no vs. yes), stroke (no vs. yes), CHD (no vs. yes), and COPD (no vs. yes). Interactions between subgroups were assessed using the likelihood ratio test.

An exploratory receiver operating characteristic (ROC) curve was performed to evaluate the ability of inflammatory/nutritional indices to predict treatment outcomes and mortality. Predictive performance was quantified using the area under the curve (AUC). Spearman correlation analysis was performed to compute the correlation coefficients and explore the relationships between inflammatory/nutritional indices and their components. Subsequently, to explore key nutritional and inflammatory markers most strongly associated with treatment outcomes in PTB patients complicated by hypertension, three machine learning algorithms were implemented using the caret package in R: Random Forest (RF), Generalized Linear Model (GLM), and Support Vector Machine (SVM) with a radial basis function kernel. The dataset was randomly partitioned (seed = 42) via stratified sampling into a training set (70%) and an independent test set (30%). All models were optimized through 10-fold cross-validation using AUC-ROC as the primary metric, with hyperparameter tuning performed via grid search (tuneLength = 5). Each final model was retrained on the entire training set before evaluation on the held-out test set. Model performance was compared using accuracy, sensitivity, specificity, precision (positive predictive value), and F1-score, with the area under the receiver operating characteristic curve (AUC-ROC) also reported to assess discrimination ability. To evaluate potential overfitting, we compared the cross-validation performance on the training set with the results on the independent test set. Permutation-based variable importance was estimated using the DALEX framework (version 2.5.2) to ensure consistent feature contribution assessment across all three models (29, 30).

All computations were executed in R (version 4.3.0; R Foundation for Statistical Computing, Vienna, Austria). A two-sided *p* value < 0.05 was considered statistically significant.

### Sensitivity analysis

To minimize overfitting given the limited number of outcome events, Model 2 was prespecified as the primary model based on events-per-variable (EPV) considerations. Additional TB- and hypertension-related clinical characteristics (Supplementary Table 3), including baseline systolic blood pressure, baseline diastolic blood pressure, sputum culture, number of lung lobes affected, duration of anti-tuberculosis treatment, adverse drug reactions to anti-tuberculosis drugs, and hypertension duration, were further adjusted in a sensitivity model (Model 3) to assess robustness (Supplementary Table 4).

## Results

### Baseline characteristics and outcomes

Overall, 1,012 hospitalized patients with untreated PTB complicated by hypertension were included in this study (Table 1). The mean age of the total population was 66.69 years, with the unfavorable outcomes group being significantly older than the favorable group (69.95 years). Among the enrolled patients, 166 (16.40%) had unfavorable TB treatment outcomes and 846 (83.60%) had favorable outcomes. Males comprised 712 (70.36%) of the study population. In terms of comorbidities, CHD and CKD were significantly more prevalent in the unfavorable outcomes group (34.34%; *p* = 0.042 and 16.27%; *p* = 0.005, respectively).

**Table 1:** Clinical characteristics of hospitalized patients with untreated PTB complicated by hypertension, stratified by treatment outcomes.

Characteristics	Total (*n* = 1,012)	Unfavorable TB treatment outcomes (*n* = 166)	Favorable TB treatment outcomes (*n* = 846)	*p*-value
Age, years	66.69 ± 11.39	69.95 ± 12.34	66.05 ± 11.09	< 0.001
Sex, *n* (%)				0.333
Male	712 (70.36)	122 (73.49)	590 (69.74)	
Female	300 (29.64)	44 (26.51)	256 (30.26)	
Education, *n* (%)				0.072
Junior high school or below	472 (46.64)	88 (53.01)	384 (45.39)	
Senior high school or above	540 (53.36)	78 (46.99)	462 (54.61)	
Smoking, *n* (%)				0.055
No	532 (52.57)	76 (45.78)	456 (53.9)	
Yes	480 (47.43)	90 (54.22)	390 (46.1)	
Drinking, *n* (%)				0.916
No	741 (73.22)	121 (72.89)	620 (73.29)	
Yes	271 (26.78)	45 (27.11)	226 (26.71)	
Stroke, *n* (%)				0.275
No	863 (85.28)	137 (82.53)	726 (85.82)	
Yes	149 (14.72)	29 (17.47)	120 (14.18)	
CHD, *n* (%)				0.042
No	730 (72.13)	109 (65.66)	621 (73.4)	
Yes	282 (27.87)	57 (34.34)	225 (26.6)	
CKD, *n* (%)				0.005
No	909 (89.82)	139 (83.73)	770 (91.02)	
Yes	103 (10.18)	27 (16.27)	76 (8.98)	
COPD, *n* (%)				0.626
No	901 (89.03)	146 (87.95)	755 (89.24)	
Yes	111 (10.97)	20 (12.05)	91 (10.76)	
Hyperlipidemia, *n* (%)				0.519
No	811 (80.14)	130 (78.31)	681 (80.5)	
Yes	201 (19.86)	36 (21.69)	165 (19.5)	
Diabetes, *n* (%)				0.509
No	617 (60.97)	105 (63.25)	512 (60.52)	
Yes	395 (39.03)	61 (36.75)	334 (39.48)	
Inflammation and nutritional indicators				
Neutrophil, ×10^3^/μL	5.18 ± 3.11	5.80 ± 4.18	5.06 ± 2.84	0.005
RDW, %	13.69 ± 1.67	14.15 ± 2.04	13.60 ± 1.57	< 0.001
Platelet count, ×10^3^/μL	248.38 ± 101.13	228.22 ± 95.16	252.33 ± 101.84	0.005
Lymphocyte, ×10^3^/μL	1.18 ± 0.59	1.07 ± 0.55	1.20 ± 0.59	0.012
Monocyte, ×10^3^/μL	0.40 (0.30, 0.56)	0.40 (0.33, 0.57)	0.40 (0.30, 0.56)	0.439
Hemoglobin, g/L	116.73 ± 22.40	108.60 ± 25.62	118.32 ± 21.37	< 0.001
Serum albumin, g/L	34.52 ± 6.07	32.60 ± 6.73	34.90 ± 5.86	< 0.001
Inflammation/nutrition-based indicators				
RAR	4.09 ± 1.16	4.55 ± 1.41	4.01 ± 1.08	< 0.001
NAR	1.27 (0.90, 1.85)	1.44 (1.04, 2.30)	1.24 (0.88, 1.77)	< 0.001
MAR	0.12 (0.09, 0.17)	0.13 (0.10, 0.19)	0.12 (0.09, 0.17)	0.038
PNI	40.40 ± 7.55	37.96 ± 8.07	40.88 ± 7.35	< 0.001
HALP	18.60 (10.38, 31.53)	15.80 (8.07, 26.08)	19.26 (10.81, 32.58)	0.007

IQR, interquartile range; SD, standard deviation. RAR, red cell distribution width-albumin ratio; NAR, neutrophil-albumin ratio; PNI, prognostic nutritional index; MAR, monocyte-albumin ratio; HALP, hemoglobin, albumin, lymphocyte, and platelet. The normality of continuous variables was assessed using histograms and the Kolmogorov–Smirnov test. Normally distributed variables are presented as mean ± SD and compared using the independent samples t-test; non-normally distributed variables are presented as median (IQR) and compared using the Mann–Whitney U test. Categorical variables are expressed as frequency (%) and compared using the χ^2^ test.

Regarding inflammation and nutritional indicators, patients with unfavorable outcomes exhibited significantly higher neutrophil counts (*p* = 0.005) and red cell distribution width (RDW) values (*p* < 0.001), but lower platelet counts (*p* = 0.005), lymphocyte counts (*p* = 0.012), hemoglobin levels (*p* < 0.001), and serum albumin levels (*p* < 0.001). Furthermore, inflammation/nutrition-based indicators including RAR, NAR, MAR, PNI, and HALP differed significantly between groups, with the unfavorable outcomes group showing higher RAR (*p* < 0.001), NAR (*p* < 0.001), and MAR values (*p* = 0.038), but lower PNI (*p* < 0.001) and HALP scores (*p* = 0.007).

Supplementary Table 3 presents additional clinical variables stratified by TB treatment outcomes, including sputum bacteriology, duration of anti-tuberculosis treatment, number of lung lobes affected, adverse reactions to anti-TB drugs, and hypertension duration. Patients with unfavorable outcomes had a shorter course of anti-tuberculosis treatment (median 6.00 vs. 12.00 months, *p* < 0.001) and a greater number of affected lung lobes (median 5.00 vs. 3.00, *p* < 0.001). Adverse drug reactions differed between groups (33.13% vs. 41.84%, *p* = 0.037).

### Association of inflammation/nutrition-based indicators with treatment outcomes among previously untreated PTB patients with hypertension

Logistic regression analysis was conducted to examine the associations between inflammation/nutrition-based indicators and favorable treatment outcome among previously untreated PTB patients with hypertension. As shown in Table 2, higher RAR was consistently associated with lower odds of favorable treatment outcomes, remaining significant after full adjustment for potential confounders (aOR: 0.75; 95% CI: 0.64–0.89; *p* = 0.001). NAR showed a similar negative association with favorable outcomes across models, with ORs of 0.40 (95% CI: 0.19–0.84; *p* = 0.015) in Model 2. MAR was associated with odds of favorable outcomes in the crude analysis (OR: 0.44; 95% CI: 0.20–0.96; *p* = 0.040), but the association was attenuated and no longer significant after multivariable adjustment (*p* = 0.370). In contrast, PNI was positively associated with favorable outcomes and remained significant in the fully adjusted model (aOR: 1.03; 95% CI: 1.01–1.06; *p* = 0.011). HALP was positively associated with favorable outcomes in the crude model (OR: 2.03; 95% CI: 1.25–3.30; *p* = 0.004), but this association did not persist after full adjustment (aOR: 1.34; 95% CI: 0.79–2.26; *p* = 0.275).

**Table 2:** Association between inflammation/nutrition-based indicators and favorable treatment outcome among previously untreated PTB patients with hypertension.

Variable	Crude		Model 1		Model 2^a^	
	OR (95%CI)	*p*-value	OR (95%CI)	*p*-value	OR (95%CI)	*p*-value
RAR	**0.67 (0.57–0.77)**	**< 0.001**	**0.70 (0.60–0.82)**	**< 0.001**	**0.75 (0.64–0.89)**	**0.001**
NAR*	**0.26 (0.13–0.52)**	**< 0.001**	**0.30 (0.15–0.61)**	**0.001**	**0.40 (0.19–0.84)**	**0.015**
MAR*	0.44 (0.20–0.96)	0.040	0.55 (0.24–1.25)	0.151	0.68 (0.29–1.58)	0.370
PNI	**1.06 (1.03–1.08)**	**< 0.001**	**1.05 (1.02–1.07)**	**< 0.001**	**1.03 (1.01–1.06)**	**0.011**
HALP*	2.03 (1.25–3.30)	0.004	1.68 (1.02–2.78)	0.041	1.34 (0.79–2.26)	0.275

OR, odds ratio; CI, confidence interval; RAR, red cell distribution width-albumin ratio; NAR, neutrophil-albumin ratio; PNI, prognostic nutritional index; MAR, monocyte-albumin ratio; HALP, hemoglobin, albumin, lymphocyte, and platelet score. Crude model: unadjusted. Model 1: adjusted for age and sex. Model 2: adjusted for age, sex, education, smoking, drinking, and major comorbidities (stroke, coronary heart disease, chronic kidney disease, chronic obstructive pulmonary disease, hyperlipidemia, and diabetes). The calculation formulas and component units for all composite indices are provided in Supplementary Table 2. ^a^Goodness of fit (Akaike Information Criterion/Hosmer-Lemeshow test *p*-value) for Model 2: RAR (886.66/0.163), NAR (891.51/0.241), MAR (897.67/0.024), PNI (891.45/0.165), and HALP (897.25/0.007). *Skewed indices (NAR, MAR, and HALP) were log-transformed before analysis. For log-transformed variables, ORs correspond to the change in odds associated with a one-unit increase on the natural log scale. Bold numbers indicate statistically significant differences at a 5% level.

In the sensitivity analyses, Supplementary Table 4 further presents the associations between inflammation/nutrition indicators and treatment outcomes after additional adjustment for baseline blood pressure, sputum bacteriology, extent of lung involvement, duration of anti-tuberculosis treatment, adverse drug reactions, and hypertension duration (Model 3). In the fully adjusted model, RAR remained significantly associated with favorable outcomes (aOR: 0.76; 95% CI: 0.63–0.92; *p* = 0.005), whereas NAR, MAR, and HALP were no longer statistically significant. PNI maintained a positive association with favorable outcomes (aOR: 1.03; 95% CI: 1.00–1.06; *p* = 0.035).

**Figure 2:**
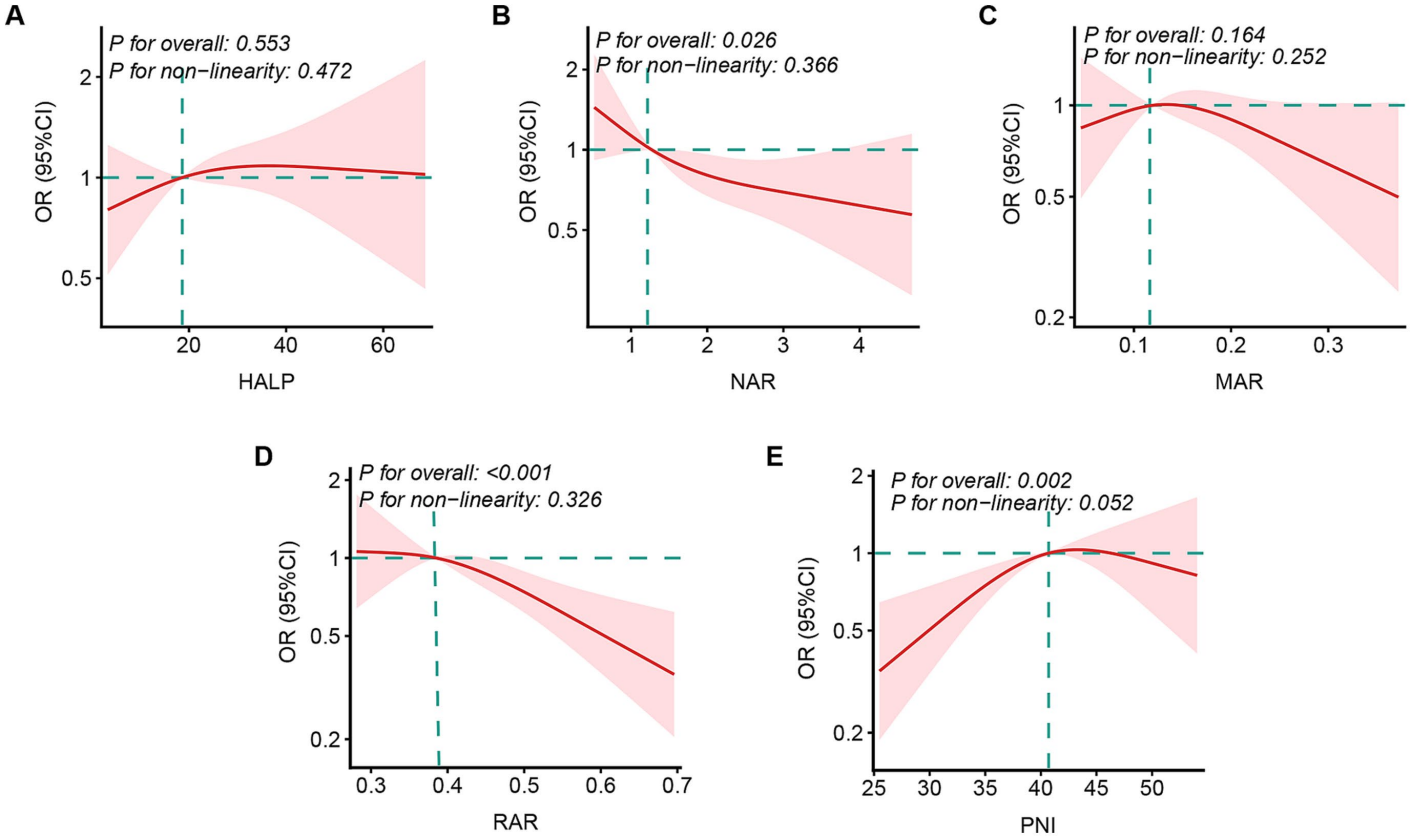
Associations between inflammation/nutrition indices and PTB treatment outcomes in patients with PTB and hypertension. Restricted cubic spline analyses were conducted to examine the dose–response relationships between inflammation-nutrition indices and treatment outcomes, including HALP **(A)**, NAR **(B)**, MAR **(C)**, RAR **(D)**, and PNI **(E)**. Models were adjusted for age, sex, educational attainment, smoking status, drinking status, stroke, coronary heart disease, chronic kidney disease, and chronic obstructive pulmonary disease. The median value of each index was used as the reference (vertical dashed line). Solid red lines represent the estimated odds ratios (ORs) for treatment outcomes, and shaded areas indicate the corresponding 95% confidence intervals (CIs). RAR, red cell distribution width-albumin ratio; NAR, neutrophil-albumin ratio; PNI, prognostic nutritional index; MAR, monocyte-albumin ratio; HALP, hemoglobin, albumin, lymphocyte, and platelet.

Restricted cubic spline models were applied to assess potential non-linear associations between inflammation/nutrition indices and the odds of treatment success (Figure 2). HALP (Figure 2A) showed no evidence of association (*p* for overall = 0.285). NAR (Figure 2B) was inversely associated with favorable treatment outcome (*p* for overall = 0.030). MAR (Figure 2C) showed no association (*p* for overall = 0.966). RAR (Figure 2D) was significantly associated with favorable treatment outcome and the association was approximately linear (*p* for overall = 0.013; *p* for non-linearity = 0.626). For PNI (Figure 2E), the association was significant (*p* for overall = 0.002) and appeared approximately non-linear (*p* for non-linearity = 0.085).

### Subgroup analyses

**Figure 3:**
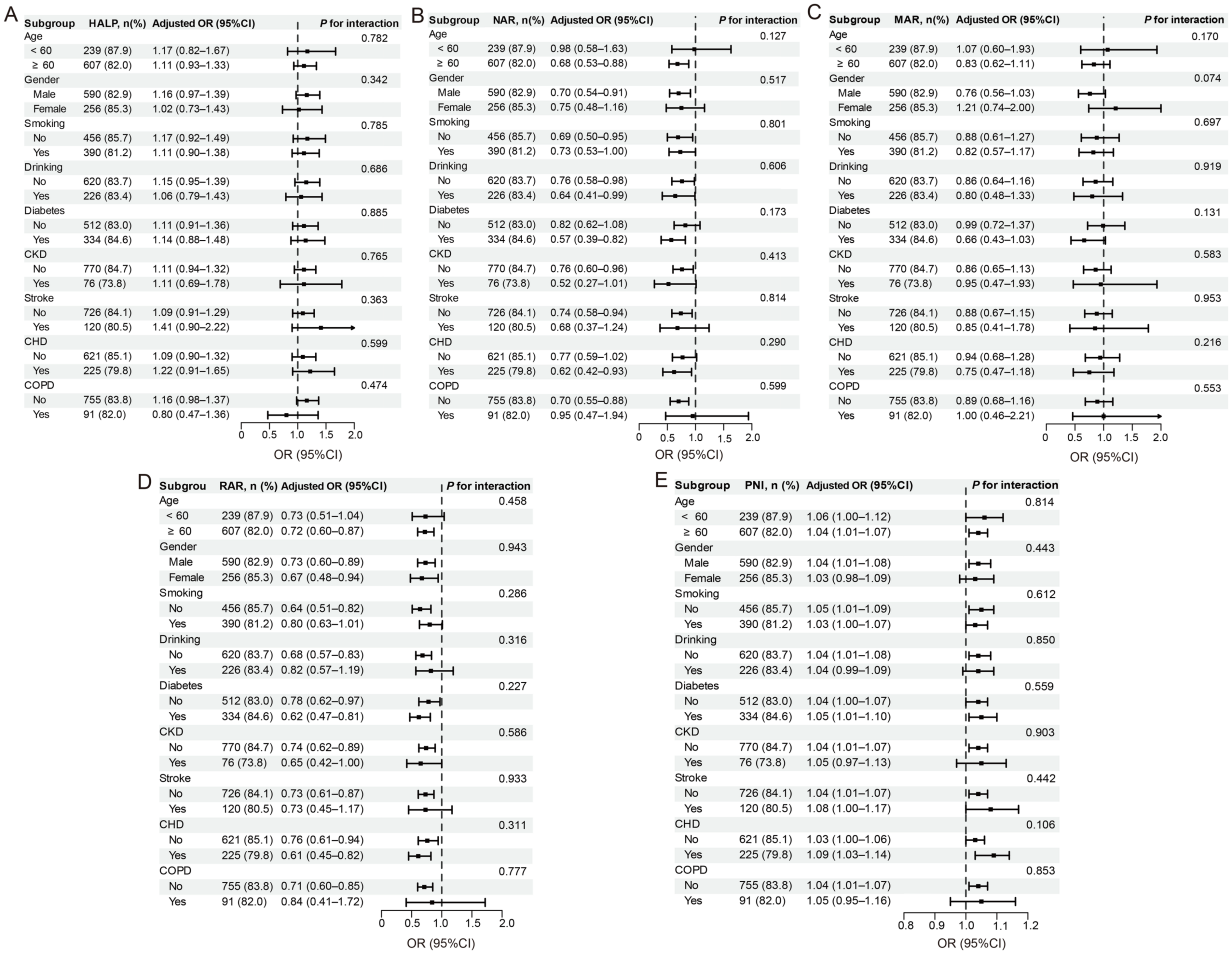
Subgroup analysis of relationship between inflammation/nutrition-based indicators and PTB treatment outcomes. Each stratification was adjusted for age, sex, education, smoking, drinking, and major comorbidities (stroke, coronary heart disease [CHD], chronic kidney disease [CKD], chronic obstructive pulmonary disease [COPD], hyperlipidemia, and diabetes) except the stratification factor itself. Squares indicate odds ratios (ORs), with horizontal lines indicating 95% CIs. **(A)**:HALP; **(B)**:NAR; **(C)**:MAR; **(D)**:RAR; **(E)**:PNI.

Figure 3 illustrates stratified subgroup analyses across age, sex, smoking status, alcohol consumption, and principal comorbidities (hyperlipidemia, diabetes mellitus, stroke, CHD, CKD, and COPD). The associations between inflammation/nutrition-based indicators and treatment outcomes in untreated pulmonary tuberculosis patients with hypertension remained consistent across all subgroups. Specifically, no significant interactions were observed between stratification variables and these inflammation/nutrition-based indices (*p* > 0.05 for interaction).

### Treatment outcomes assessment using inflammation/nutrition-based indicators and their components

**Figure 4:**
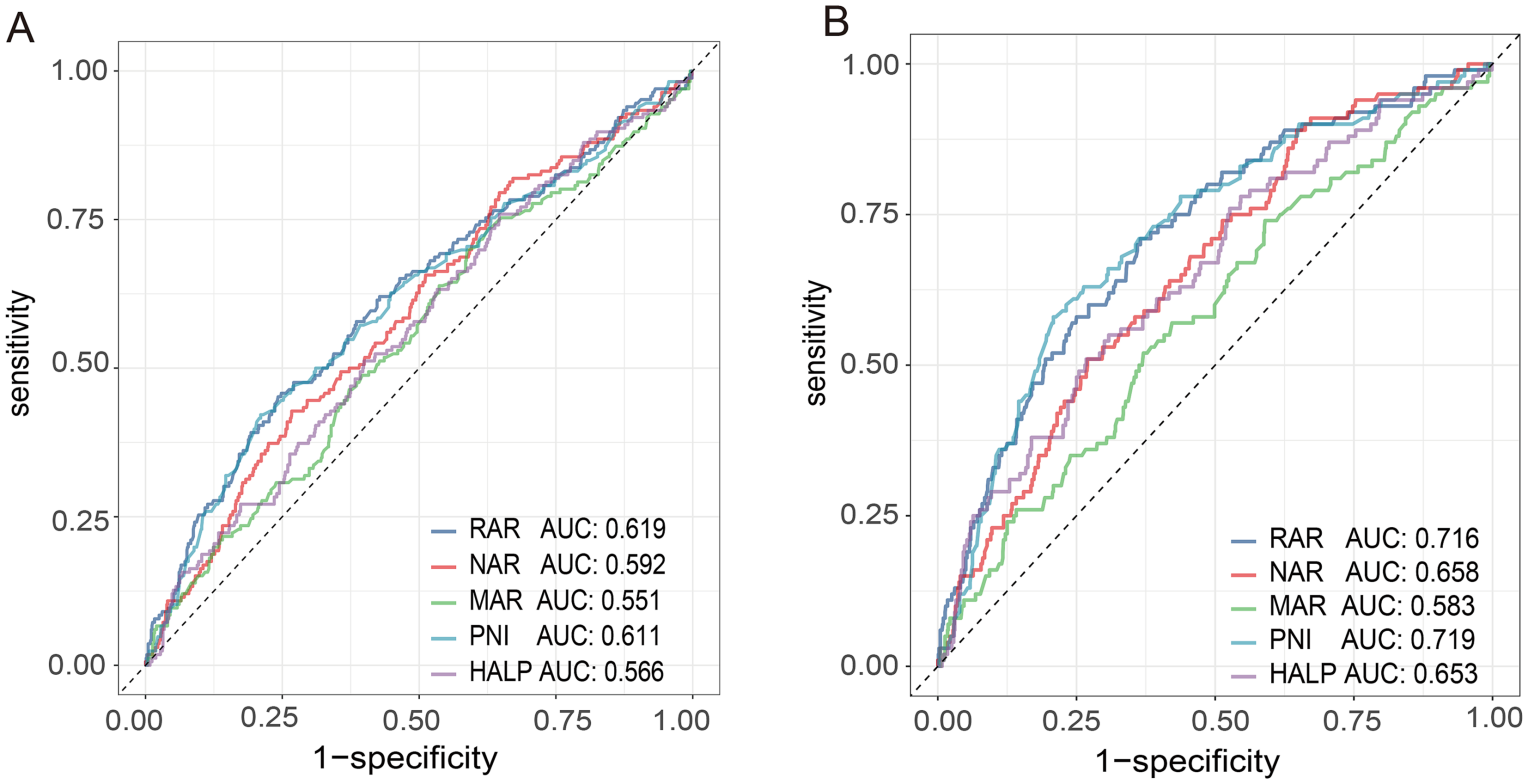
ROC analysis for predicting unfavorable treatment outcomes **(A)** and mortality **(B)** using inflammation/nutrition-based indicators (RAR, NAR, MAR, PNI, and HALP) in hypertension with untreated pulmonary tuberculosis patients. RAR, red cell distribution width-albumin ratio; NAR, neutrophil-albumin ratio; MAR, monocyte-albumin ratio; PNI, prognostic nutritional index; HALP, hemoglobin, albumin, lymphocyte, and platelet.

**Figure 5:**
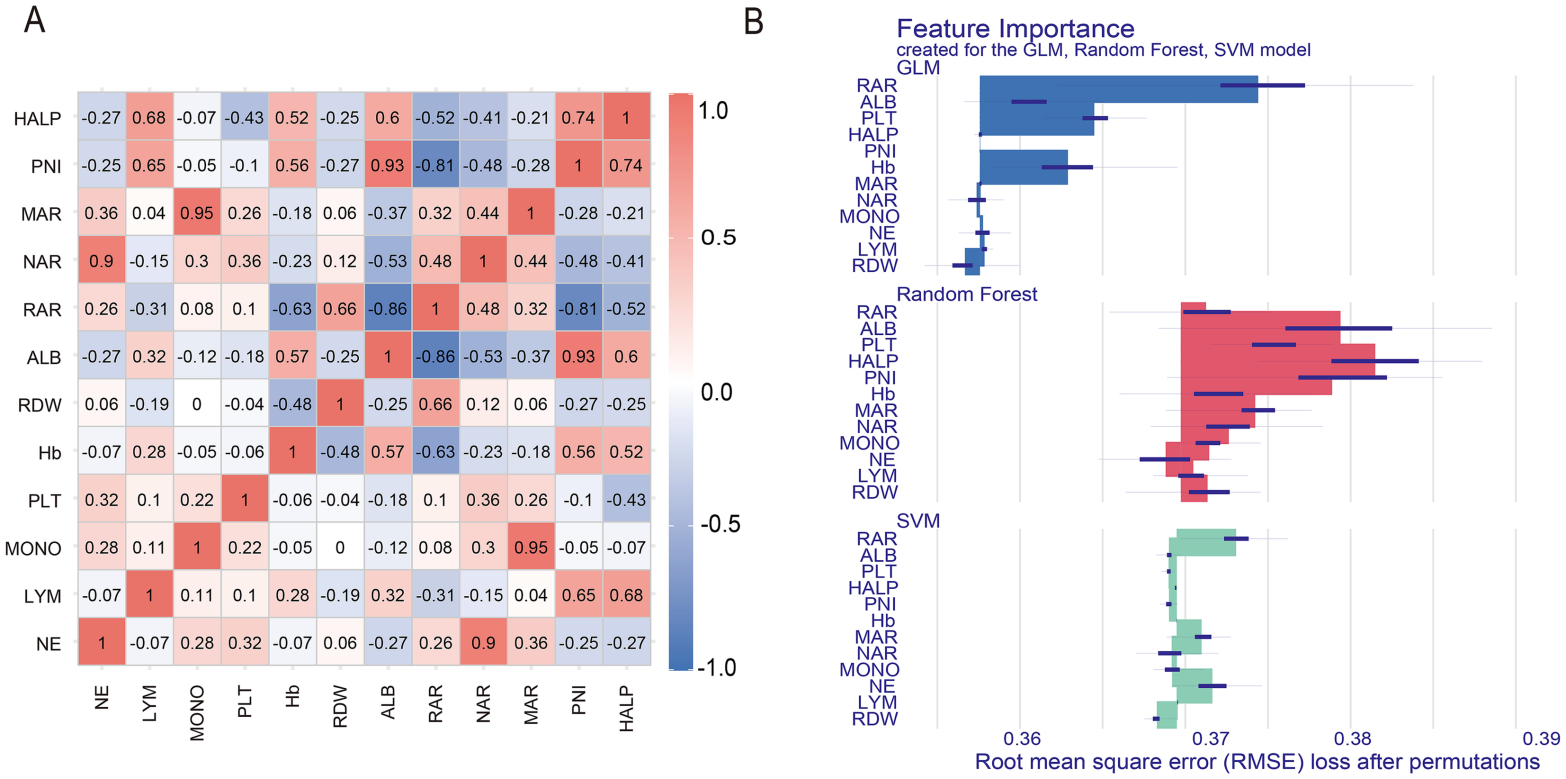
Importance of inflammatory and nutritional indicators, along with their components, for treatment outcomes in hypertensive patients with untreated pulmonary tuberculosis. **(A)** The correlation relationship between inflammatory/nutrition-based indicators and their components was determined through Spearman’s correlation testing; **(B)** The relative contributions of these markers to treatment outcomes were ranked via generalized linear model (GLM), random forest (RF), and support vector machine (SVM) machine learning algorithms. RAR, red cell distribution width-albumin ratio; NAR, neutrophil-albumin ratio; MAR, monocyte-albumin ratio; PNI, prognostic nutritional index; HALP, hemoglobin, albumin, lymphocyte, and platelet. NE, neutrophil; LYM, lymphocyte; MONO, monocyte; Hb, hemoglobin; RDW, red cell distribution width; ALB, albumin; PLT, platelet count.

Receiver operating characteristic (ROC) curves were used to assess the ability of inflammation/nutrition-based indicators to differentiate treatment outcome groups among previously untreated PTB patients with concurrent hypertension. The ROC analysis showed that RAR and PNI demonstrated comparatively higher AUC values than the other indices, with AUCs of 0.619 and 0.611, respectively (Figure 4A). In addition, both markers exhibited better classification ability for all-cause mortality, with AUC values of 0.716 for RAR and 0.719 for PNI (Figure 4B). Despite this, further combination of RAR and PNI into a joint model yielded no improvement in discrimination over RAR alone (AUC: 0.619 vs. 0.619; DeLong test: *p* = 0.836), with NRI and IDI also non-significant (Supplementary Figure 1). Furthermore, Spearman’s correlation analysis uncovered distinct interrelationships between these composite inflammation/nutrition indices and their individual components (Figure 5A). The correlation matrix revealed varying levels of interdependence among the markers, with coefficients spanning from −0.86 to 0.95.

Supplementary Table 5 shows the ROC curve analysis evaluating the predictive performance of inflammation/nutrition indicators for treatment outcomes. RAR demonstrated the highest discriminative ability (AUC: 0.619; 95% CI: 0.568–0.667), followed by PNI (AUC: 0.611; 95% CI: 0.562–0.661) and NAR (AUC: 0.592; 95% CI: 0.544–0.639), whereas MAR and HALP showed lower performance (AUCs: 0.551 and 0.566, respectively). Optimal cut-offs were 4.532 (RAR), 34.775 (PNI), 1.696 (NAR), 0.106 (MAR), and 15.836 (HALP), with Youden indices ranging from 0.111 to 0.211.

To further explore the relative importance of these variables, variable importance ranking was conducted using three machine learning-based exploratory approaches—generalized linear model (GLM), random forest (RF), and support vector machine (SVM) (Figure 5B). Among the evaluated variables, RAR exhibited the highest importance across the applied models. Supplementary Table 6 summarizes the performance metrics: The GLM model outperformed the others with the highest AUC-ROC (0.612, 95% CI 0.528–0.696), accuracy (0.822), and F1-score (0.901), while all models demonstrated high sensitivity but low specificity.

## Discussion

This study investigated the associations between inflammation/nutrition-based indicators and treatment outcomes in previously untreated PTB patients with hypertension. A total of 166 patients (16.40%) experienced unfavorable outcomes. Multivariable logistic regression analyses demonstrated that RAR and NAR were inversely associated with favorable treatment, whereas PNI exhibited a positive association. Conversely, MAR and HALP lost statistical significance following comprehensive covariate adjustment. Restricted cubic spline modeling confirmed an approximately linear relationship for RAR and significant overall associations for NAR and PNI. Our findings underscore the significance of RAR and PNI, which demonstrated the highest AUC among the indicators for treatment outcome compared to the remaining indicators. Machine learning feature-importance rankings across GLM, RF, and SVM models consistently identified RAR as the strongest contributor across applied models. These findings underscore the potential clinical utility of integrating inflammatory and nutritional markers.

### Comparison with prior literature

Composite inflammatory-nutritional indices, including RAR, NAR, MAR, PNI, and HALP, have been explored in infectious diseases. For instance, Tan et al. (31) analyzed a cohort of patients with newly diagnosed PTB and found that low PNI (cut-off 39.825) was a strong independent predictor of treatment failure or poor prognosis (adjusted OR = 23.667, 95% CI: 9.317–60.115, *p* < 0.0001); patients with comorbidities such as diabetes and COPD were more likely to exhibit elevated malnutrition. While the HALP score is traditionally used to assess mortality risk (HR: 3.970, 95% CI: 1.622–9.485) in complex TB cases (32). Furthermore, although NAR has been preliminarily applied to monitor anti-tuberculosis efficacy, research within the pathophysiological context of hypertension remains scarce (33). Notably, systematic investigations exploring the clinical utility of RAR and MAR remain sparse, particularly when focusing on PTB patients affected by chronic comorbidities. Our study extends these findings by simultaneously evaluating multiple indices in hospitalized, previously untreated PTB patients with hypertension. This population represents a high-risk group characterized by chronic low-grade inflammation and endothelial dysfunction. We employed a composite outcome that includes treatment failure, recurrence, drug resistance, or death. Incorporating RAR, NAR, MAR, and HALP provides a more integrated assessment of nutritional and inflammatory status, addressing limitations of a single parameter. These results offer observational evidence of correlations in a high-risk TB subgroup, while emphasizing that causality cannot be inferred.

In our study, RAR and NAR were negatively associated with favorable outcomes, which is consistent in interpretation with prior studies showing that higher inflammation-albumin composite indices predict worse prognosis in infectious and inflammatory diseases (34, 35). That is, elevated RAR or NAR generally reflects either a higher inflammatory burden, lower albumin, or both, corresponding to a higher risk of adverse outcomes. However, the current findings should be interpreted in context with caution. First, we used a composite outcome including treatment failure, recurrence, drug resistance, and death rather than mortality alone, which means a more complex outcome. Second, our study population consisted of hospitalized, previously untreated PTB patients with hypertension, a group prone to chronic low-grade inflammation, endothelial dysfunction, and altered protein metabolism. These factors may affect the associations between RAR/NAR and treatment outcomes. Third, residual confounding from unmeasured variables such as TB severity, nutritional interventions, and comorbidity burden may influence the observed associations.

The association of higher PNI with favorable outcomes aligns with most previous investigations: low PNI reflects malnutrition and immunosuppression, both of which may worsen TB by impairing cell-mediated immunity (36, 37). Importantly, in the context of PTB complicated by hypertension, PNI may additionally capture the combined effects of nutritional status and vascular-endothelial integrity. Hypertension-related endothelial dysfunction can exacerbate systemic inflammation and protein-energy wasting, which may modify the prognostic relevance of PNI (11, 38). Thus, higher PNI likely reflects better physiologic reserve and preserved microvascular function, contributing to favorable outcomes. Nevertheless, the attenuation of associations for MAR and HALP after adjustment further indicates sensitivity of these indices to comorbid conditions, medication exposure, and disease severity.

Compared with our previous study (14), which focused solely on mortality, the current study analyzed a composite outcome encompassing treatment failure, disease recurrence, drug resistance, and death. While mortality represents an extreme and relatively infrequent endpoint, composite outcomes capture a broader spectrum of clinically relevant events that reflect treatment effectiveness and disease progression. Studying these composite outcomes in hospitalized PTB patients with hypertension is particularly important, as this population may experience multiple adverse events that do not result in immediate death but still indicate disease severity and treatment challenges. Notably, there are no prior studies specifically addressing the relationship between inflammatory/nutritional indices and prognosis in PTB patients with coexisting hypertension, highlighting the novelty and clinical relevance of our study. It is also a further extension of previous studies. By focusing on this unique patient subgroup, our study addresses a gap in the literature and provides initial evidence for the utility of RAR, NAR, and PNI in this population.

ROC curve analysis revealed modest AUC values for inflammatory-nutritional indicators (maximum 0.719), with slightly better discrimination for mortality than for composite unfavorable outcomes. Additionally, our ML exploration showed statistically significant associations for the GLM model. However, the very low specificity (< 6%) across all models indicates that these indicators cannot reliably identify patients at risk of unfavorable outcomes. These findings suggest that RAR, NAR, and PNI may provide potential reference information for risk stratification but should not be considered definitive clinical tools. Further prospective studies with repeated biomarker measurements, stratified by comorbidity and treatment exposure, are needed to validate their utility.

### Clinical implications

The associations between RAR, NAR, PNI and treatment outcomes hold important clinical relevance for resource-limited TB control settings. These composite indices, derived from routine laboratory parameters, enable cost-effective risk stratification at treatment initiation. Patients with elevated RAR or reduced PNI values may warrant intensified monitoring and early nutritional intervention. Furthermore, machine-learning models incorporating multiple inflammation-nutrition markers demonstrated enhanced feature importance for RAR, suggesting that integrated inflammation-nutrition indices could serve as exploratory references in risk stratification and clinical management of high-risk populations, pending validation in prospective studies.

### Limitations

Despite our findings, several limitations should be acknowledged. First, the retrospective, single-center design may have selection bias and residual confounding from unmeasured variables, including TB severity, medication exposures, and socioeconomic factors. Second, reliance on baseline rather than longitudinal measurements may underestimate the prognostic value of inflammatory-nutritional indices, and the composite outcome may obscure endpoint-specific associations. Third, systematic immunologic testing (e.g., CD4 + T-cell counts) was not universally available in this EMR-based cohort, potentially leading to misclassification of immunocompromised status and limiting external validity. Fourth, key confounders, including antihypertensive drug class and anti-tuberculosis therapy adherence, were insufficiently captured and may have distorted observed associations. Although the Hosmer-Lemeshow test indicated suboptimal calibration for certain variables, this does not compromise our primary objective of identifying independent associations rather than developing a predictive model. Importantly, all observed associations are correlational and should not be interpreted as causal. Future prospective, multicenter studies should incorporate systematic documentation of anti-tuberculosis regimens (e.g., 2, 6, or 12 months after anti-tuberculosis therapy), standardized adherence assessment, and serial measurement of nutritional and inflammatory indices. Repeated assessment of these markers at multiple treatment time points could provide valuable insights into their dynamic relationship with treatment response and reveal more accurate associations with patient outcomes.

## Conclusion

This study demonstrates that routine laboratory-derived composite indices, particularly RAR and PNI, are significantly associated with treatment outcomes in previously untreated PTB patients with hypertension. In resource-limited settings, these accessible parameters may provide potential reference indicators for risk stratification. However, prospective studies are needed to verify the optimal cut-off values and clinical application value.

## Data Availability

The datasets presented in this article are not readily available because the original contributions presented in the study are included in the article/Supplementary material. Further inquiries can be directed to the corresponding author. Requests to access the datasets should be directed to Yiping Leng, lyp0626@aliyun.com.
