# Supplementary material for: Prognostic significance of inflammatory and nutritional indicators for treatment outcomes in untreated tuberculosis patients with hypertension
Source: Front Nutr. 2026 Apr 22;13:1801389. doi: 10.3389/fnut.2026.1801389 (PMC13143731; doi:10.3389/fnut.2026.1801389)
Supplement: Supplementary file 1 [file Table_1.docx]

Online Supplementary Material

**Prognostic significance of inflammatory and nutritional indicators for treatment outcomes in untreated tuberculosis patients with hypertension**

**Materials and methods:** *Assessment of inflammation and nutritional indicators*

Complete blood count (CBC) tests in this study were performed using an automated hematology analyzer (Sysmex XN-9000). All analyses were conducted in accordance with standard operating procedures, and internal quality control was performed daily using analyzer-specific quality control materials as part of the laboratory’s internal quality assurance program. Differential leukocyte counts were determined using fluorescence flow cytometry following erythrocyte lysis and nucleic acid staining, with cell populations classified based on light scatter and fluorescence signals. Platelet (PLT) enumeration was primarily conducted using the direct current (DC) sheath flow impedance method (PLT-I) on the automated hematology analyzer. Serum albumin (ALB) was measured using the bromocresol green (BCG) dye-binding method on a Hitachi 7600 automatic biochemical analyzer (Hitachi High-Technologies Corporation, Tokyo, Japan). In this method, albumin binds to bromocresol green at pH 4.2 to form a colored complex, and the absorbance is measured spectrophotometrically at 628 nm; the intensity of color produced is directly proportional to the albumin concentration in the sample.

All instrument calibrations were carried out using appropriate calibration reagents to ensure analytical precision and accuracy. The calibration process aimed to align measured parameter values with the manufacturer-recommended target values and reference ranges. In addition, the clinical laboratory (certified ISO15189) regularly participated in external quality assessment (EQA) programs organized by national or regional proficiency testing agencies. Laboratory performance was periodically evaluated through blinded analysis of standardized samples, and results were required to meet predefined acceptance criteria to ensure inter-laboratory comparability and analytical reliability.

**Table S1** Normal reference ranges for inflammation-nutrition indicators in adults (≥ 18 years)

| **Indicator** | **Unit** | **Male** | **Female** |
| --- | --- | --- | --- |
| Neutrophil count | ×10³/μL | 1.80–6.30 | 1.80–6.30 |
| Lymphocyte count | ×10³/μL | 1.10–3.20 | 1.10–3.20 |
| Monocyte count | ×10³/μL | 0.10–0.60 | 0.10–0.60 |
| Platelet count | ×10³/μL | 125.00–350.00 | 125.00–350.00 |
| Hemoglobin | g/L | 130–175 | 115–150 |
| RDW | % | 7.00–18.00 | 7.00–18.00 |
| Serum albumin | g/L | 40.00–55.00 | 40.00–55.00 |

RDW, red cell distribution. Complete blood count reference ranges apply to adults aged ≥ 18 years and were obtained from the laboratory of Changsha Central Hospital Affiliated with the University of South China using the Sysmex XN-9000 automated hematology analyzer (Sysmex Corporation, Kobe, Japan) and standard biochemical methods. Serum albumin reference range was established using the bromocresol green (BCG) dye-binding method on a Hitachi 7600 automatic biochemical analyzer (Hitachi High-Technologies Corporation, Tokyo, Japan).

**Table S2** Definitions and calculation formulas of nutrition-inflammation composite indices

| Indicator | Calculation Formula |
| --- | --- |
| PNI | Albumin (g/L) + 5 × Lymphocyte count (×10⁹/L) |
| MAR | Monocyte count (×10⁹/L)/Albumin (g/L) |
| NAR | Neutrophil count (×10^9^/L)/Albumin (g/L) |
| RAR | 10 × Red cell distribution width (%)/Albumin (g/L) |
| HALP | [Hemoglobin (g/L) × Albumin (g/L) × Lymphocyte count (×10⁹/L)]/Platelet count (×10⁹/L) |
| RAR, red cell distribution width-albumin ratio; NAR, neutrophil-albumin ratio; PNI, prognostic nutritional index; MAR, monocyte-albumin ratio; HALP, hemoglobin, albumin, lymphocyte, and platelet. | |

**Table S3** Baseline characteristics of additional variables stratified by TB treatment outcomes

| **Characteristics** | **Total** **(n = 1,012)** | **Unavorable TB** **treatment** **outcomes (n = 166)** | **Favorable TB** **treatment** **outcomes (n = 846)** | ***p-value*** |
| --- | --- | --- | --- | --- |
| **Age (years), mean ± SD** | 66.69 ± 11.39 | 69.95 ± 12.34 | 66.05 ± 11.09 | < 0.001 |
| **Sex, n (%)** |  |  |  | 0.333 |
| Male | 712 (70.36) | 122 (73.49) | 590 (69.74) |  |
| Female | 300 (29.64) | 44 (26.51) | 256 (30.26) |  |
| **Systolic blood pressure (mmHg), mean ± SD** | 140.45 ± 20.13 | 141.13 ± 24.00 | 140.32 ± 19.29 | 0.634 |
| **Diastolic blood pressure (mmHg), mean ± SD** | 82.70 ± 13.13 | 82.02 ± 14.35 | 82.84 ± 12.88 | 0.465 |
| **Sputum bacteriology, n (%)** |  |  |  | 0.305 |
| Negative | 487 (48.22) | 74 (44.58) | 413 (48.93) |  |
| Positive | 523 (51.78) | 92 (55.42) | 431 (51.07) |  |
| **Duration of anti-tuberculosis treatment (months), median (IQR)** | 12.00 (6.00–12.00) | 6.00 (1.00–12.00) | 12.00 (9.00–12.00) | < 0.001 |
| **Number of lung lobes affected, median (IQR)** | 4.00 (2.00–6.00) | 5.00 (3.00–6.00) | 3.00 (2.00–6.00) | < 0.001 |
| **Adverse reactions to anti-TB drugs, n (%)** |  |  |  | 0.037 |
| No | 603 (59.59) | 111 (66.87) | 492 (58.16) |  |
| Yes | 409 (40.41) | 55 (33.13) | 354 (41.84) |  |
| **Hypertension duration (years), median (IQR)** | 6.00 (2.00, 10.00) | 7.50 (2.25, 10.00) | 6.00 (2.00, 10.00) | 0.157 |

IQR, interquartile range; SD, standard deviation. Continuous variables with normal distribution are presented as mean ± SD; non-normally distributed continuous variables are presented as median (IQR). Categorical variables are expressed as frequency (%).

**Table S4** Associations between inflammation/nutrition indicators and treatment outcomes in patients with previously untreated pulmonary tuberculosis complicated by hypertension

| Variable | Crude | | Model 1 | | Model 2 | | Model 3 | |
| --- | --- | --- | --- | --- | --- | --- | --- | --- |
|  | OR (95%CI) | *p*-value | OR (95%CI) | *p*-value | OR (95%CI) | *p*-value | OR (95%CI) | *p*-value |
| **RAR** | 0.67 (0.57–0.77) | **<0.001** | 0.70 (0.60–0.82) | **<0.001** | 0.75 (0.64–0.89) | **0.001** | 0.76 (0.63–0.92) | **0.005** |
| **NAR**^*^ | 0.26 (0.13–0.52) | **<0.001** | 0.30 (0.15–0.61) | **0.001** | 0.40 (0.19–0.84) | **0.015** | 0.56 (0.23–1.37) | 0.208 |
| MAR^*^ | 0.44 (0.20–0.96) | **0.040** | 0.55 (0.24–1.25) | 0.151 | 0.68 (0.29–1.58) | 0.370 | 0.68 (0.25–1.84) | 0.449 |
| **PNI** | 1.06 (1.03–1.08) | **<0.001** | 1.05 (1.02–1.07) | **<0.001** | 1.03 (1.01–1.06) | **0.011** | 1.03 (1.00–1.06) | **0.035** |
| HALP^*^ | 2.03 (1.25–3.30) | 0.004 | 1.68 (1.02–2.78) | 0.041 | 1.34 (0.79–2.26) | 0.275 | 1.17 (0.62–2.21) | 0.632 |

Abbreviations: OR, odds ratio; CI, confidence interval; RAR, red cell distribution width-to-albumin ratio; NAR, neutrophil-to-albumin ratio; MAR, monocyte-to-albumin ratio; PNI, prognostic nutritional index; HALP, hemoglobin, albumin, lymphocyte, and platelet score. Crude: unadjusted model. Model 1: adjusted for age and sex. Model 2: adjusted for age, sex, education, smoking, drinking, and major comorbidities (stroke, coronary heart disease, chronic kidney disease, chronic obstructive pulmonary disease, hyperlipidemia, and diabetes). Model 3: Model 2 + baseline systolic blood pressure, baseline diastolic blood pressure, sputum culture, number of lung lobes affected, duration of anti-tuberculosis treatment, adverse drug reactions to anti-tuberculosis drugs, and hypertension duration. *Log-transformed prior to analysis. For log-transformed variables, ORs correspond to the change in odds associated with a one-unit increase on the natural log scale. Bold values indicate *p* < 0.05.


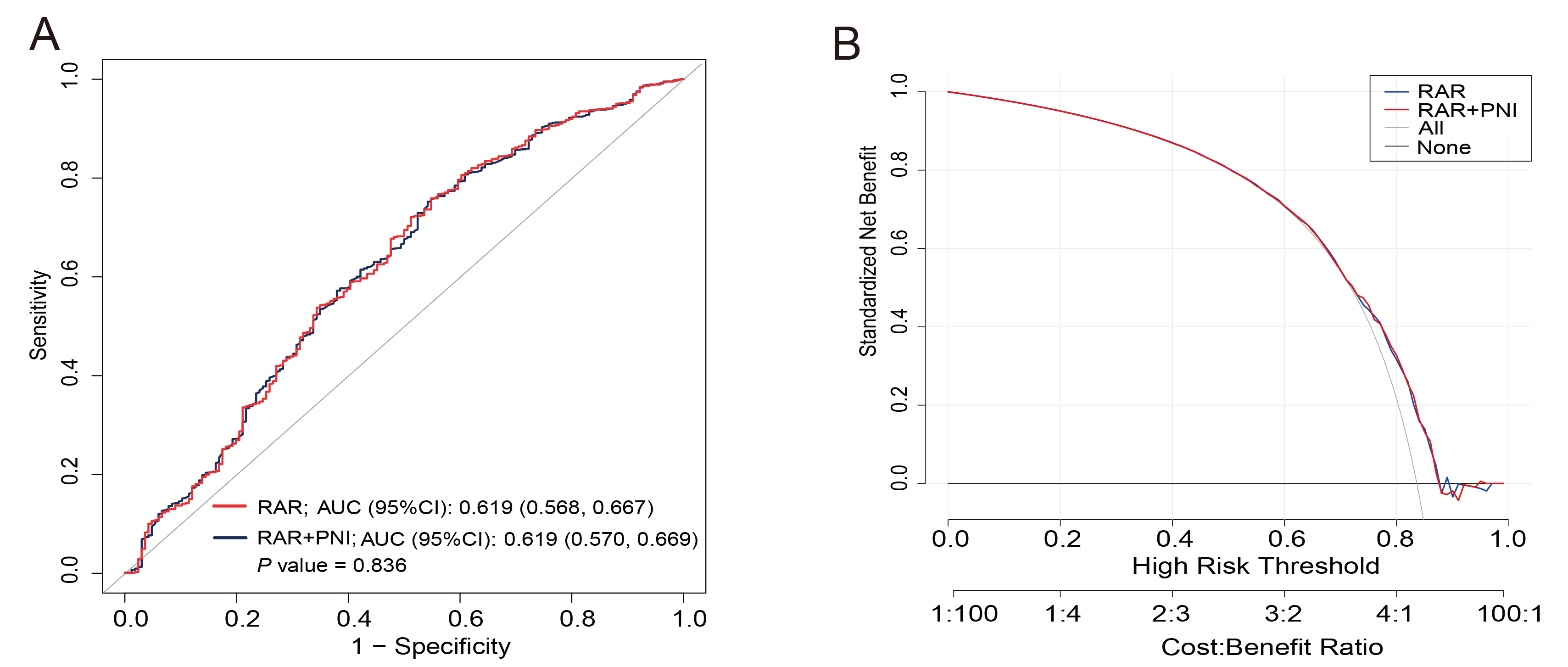


**Figure S1** Comparative predictive performance of RAR alone versus RAR combined with PNI for predicting unfavorable treatment outcomes. (A) ROC analysis of RAR, and combination of RAR and PNI for predicting unfavorable treatment outcomes. (B) Decision curve analysis illustrating the standardized net benefit of RAR alone and the combined RAR + PNI model across varying threshold probabilities. The x-axis represents the high-risk threshold probability and the y-axis represents the standardized net benefit.

**Table S5** ROC curve analysis of inflammation/nutrition indicators and treatment outcomes in patients with previously untreated PTB complicated by hypertension

| Variables | AUC (95%CI) | Cut off | Sensitivity (%) | Specificity (%) | Youden index |
| --- | --- | --- | --- | --- | --- |
| RAR | 0.619 (0.568–0.667) | 4.532 | 75.414 | 45.181 | 0.206 |
| PNI | 0.611 (0.562–0.661) | 34.775 | 78.960 | 42.169 | 0.211 |
| NAR | 0.592 (0.544–0.639) | 1.696 | 73.286 | 42.771 | 0.161 |
| MAR | 0.551 (0.502–0.600) | 0.106 | 41.253 | 69.880 | 0.111 |
| HALP | 0.566 (0.518–0.614) | 15.836 | 60.047 | 51.205 | 0.112 |

Abbreviations: ROC, receiver operating characteristic; AUC, area under the curve; CI, confidence interval; HALP, hemoglobin-albumin-lymphocyte-platelet score; NAR, neutrophil-to-albumin ratio; MAR, monocyte-to-albumin ratio; RAR, RDW-to-albumin ratio; PNI, prognostic nutritional index.

**Table S6** Performance metrics of the three machine learning models on the independent test set

| Model | AUC  (95% CI) | *p*-value | Accuracy | Sensitivity | Specificity | PPV | NPV | F1-  Score |
| --- | --- | --- | --- | --- | --- | --- | --- | --- |
| Random Forest | 0.515 (0.424–0.606) | 0.365 | 0.791 | 0.934 | 0.132 | 0.832 | 0.304 | 0.88 |
| GLM | 0.612 (0.528–0.696) | 0.005 | 0.822 | 0.988 | 0.057 | 0.828 | 0.500 | 0.901 |
| SVM | 0.554 (0.464–0.644) | 0.108 | 0.811 | 0.975 | 0.057 | 0.826 | 0.333 | 0.895 |

Abbreviations: AUC, area under the receiver operating characteristic curve; PPV, positive predictive value; NPV, negative predictive value. All metrics were evaluated on the independent test set.

**Table S7** Cross-validation and independent test set performance of three classification models

| **Model** | **Cross-validation (training set)** | | **Independent test set** | | **ΔAUC** |
| --- | --- | --- | --- | --- | --- |
|  | **AUC** | **95% CI** | **AUC** | **95% CI** |  |
| Random Forest | 0.637 | 0.412–0.862 | 0.515 | 0.424–0.606 | 0.122 |
| GLM | 0.641 | 0.499–0.784 | 0.612 | 0.528–0.696 | 0.029 |
| SVM | 0.594 | 0.343–0.846 | 0.554 | 0.464–0.644 | 0.040 |

Abbreviations: AUC, area under the receiver operating characteristic curve; CI, confidence interval; GLM, generalized linear model; SVM, support vector machine. ΔAUC = CV AUC - Test AUC; larger positive values indicate greater overfitting. 95% CIs were estimated by bootstrapping with 1,000 resamples.
